# Factors associated with early age at menarche among Thai adolescents in Bangkok: A cross-sectional study

**DOI:** 10.1186/s12905-017-0371-5

**Published:** 2017-03-09

**Authors:** Pongsak Noipayak, Petch Rawdaree, Busaba Supawattanabodee, Sumonmal Manusirivitthaya

**Affiliations:** 1 Department of Pediatrics, Faculty of Medicine, Vajira Hospital, Samsean Road, Dusit District, Bangkok, 10300 Thailand; 2 Department of Medicine, Faculty of Medicine Vajira Hospital, Navamindradhiraj University, Bangkok, 10300 Thailand; 3 Research Promoting Center, Faculty of Medicine Vajira Hospital, Navamindradhiraj University, Bangkok, 10300 Thailand

**Keywords:** Early puberty, Factors associated, Thai urban adolescents, Age at menarche, Bangkok, Thailand

## Abstract

**Background:**

The age at menarche in the Thai population has not been determined since 1997. This study recruited adolescents in Bangkok Metropolis to determine the age at menarche and its associations with health and socioeconomic status.

**Methods:**

This cross-sectional study used a two-step stratified sampling strategy to recruit 1,020 female students, aged 10–16 years, from schools in Dusit district, Bangkok, Thailand. Self-reported data on age at menarche and social determinants of health were collected from participants and their parents. A trained research nurse collected anthropometric data.

**Results:**

Mean age at menarche was 11.8 ± 1.0 years, and age at menarche was significantly correlated with year of birth (*r* = −0.4, *p* < 0.001). Students from schools that are part of Bangkok Metropolis had the lowest mean age at menarche. Participants born in 2000–2003 having their first period at < 11.8 years numbered 5.5 times (95% CI: 3.80–8.18) and 5.0 times (95% CI: 3.6–8.0) greater than those born in 1997–1999 by univariate and multivariate analysis, respectively. Year of birth significantly associated with age at menarche in univariate and multivariate analysis (*p* = 0.001).

**Conclusion:**

The mean age at menarche among female adolescents in Bangkok Metropolis was occurring earlier and was inversely associated with year of birth in this cohort. Only year of birth were associated with age at menarche in the multivariate regression models to adjust for potential confounders.

## Background

Puberty is now occurring at a markedly younger age than a century ago [1]. A great deal of research on early puberty has been conducted in girls, because puberty is easily identified by menarche. In northern Europe and the United States, the mean age at menarche has decreased by about 3 years over the last century [2–6]. Similarly, a 2008 study in India reported that age at menarche had declined by approximately 7 months per decade since 1960 [7]. In Thailand, a study conducted in 1987 reported that the mean age at menarche in Bangkok Metropolis was 12.2 years [8], and a later study, conducted in 1997, revealed that the age at menarche among obese adolescents was 12.5 years [9]. Apart from genetic influences [10, 11], the global trend of an earlier age at menarche is considered a consequence of improved nutrition [10], reduced infection rates [10], exposure to chemical substances [11], and environmental factors [12]. Earlier age at menarche has been associated with negative behavioral [1] and health outcomes, such as breast cancer [13], type 2 diabetes, dyslipidemia, and cardiovascular disease [14, 15].

The age at menarche in the Thai population has not been reported since 1997. In addition, the 1997 survey was on obese adolescents and the results were not representative of the population as a whole. The purpose of this study was to establish age at menarche in Thailand in a population-based study and to investigate associations between social determinants of health and age at menarche.

## Methods

This study is part of the Dusit Study Project, conducted from June to December 2013. The Dusit Study Project was a cross-sectional study investigating female pubertal onset and its associations with social determinants of health, as well as behavioral problems and intellectual quotient. It focused on female students in grades 5–9 in Bangkok’s Dusit District. The age at menarche and intelligence quotients has already been published elsewhere [16]. This paper will demonstrate factors associated with early age at menarche which have not been mentioned in the previous paper. This work was approved by the Faculty of Medicine’s Committee on Research Ethics at Vajira Hospital, Navamindradhiraj University (University of Bangkok Metropolis), in Bangkok, on July 30, 2012.

The sample size was calculated using an alpha error of 0.05 and a mean age at menarche of 12.62 ± 1.05 years from a study conducted in India in 2007 in an Asian urban population [7]. As a result, 848 participants were required. The sample size was increased by 20% to increase the statistical power and to compensate for non-respondents or a large number of participants who had not yet experienced menarche. Therefore, a target 1,018 female participants was set.

A two-step stratified sampling strategy was employed to recruit participants. In the first step, all 17,045 female students from 47 schools in Dusit District were classified into four groups according to their schools: (1) those affiliated with Bangkok Metropolis (Bangkok Metropolis schools); (2) those affiliated with the office of the Basic Education Commission of Thailand (Ministry of Education schools); (3) teacher-training demonstration schools (demonstration schools); and (4) those affiliated with the office of the Private Education Commission (private schools). Next, a school or schools from each of the four groups was selected via simple random sampling: two mixed-sex schools from Bangkok Metropolis schools, one mixed-sex Ministry of Education school, one mixed-sex demonstration school, and two private girls’ schools. In the second step, clusters of students by classroom were sampled to maximize the convenience of data collection. Students who did not receive parental permission to participate, those with a history of illnesses involving sex steroids, such as pituitary tumors or steroid treatment, and those who were unable to read and/or write were excluded. Finally, 1,020 participants were enrolled.

Questionnaires and informed consent forms were sent to students and their parents via teachers and then collected within a week. The questionnaires were used to gather self-report data from students on age at menarche; social determinants of health, such as exercise frequency, year of birth, BMI, type of school, parents’ age at birth, family income, food intake and preferences, leisure activities, monetary allowance, and the amount of time spent sleeping; amount of time spent playing video games; amount of time spent watching television; amount of time spent reading; and employment in a part time job. Parents also filled out questionnaires on demographic data. Questionnaires that were not returned after a participant had received two telephone calls, 3 days apart, during the second week after survey distribution were defined as non-responders. Finally, a trained research nurse performed anthropometric measurements, including weight, height, and waist circumference following the Centers for Disease Control and Prevention (CDC) standard [17].

During the course of fieldwork, there were some unforeseen deviations from the protocol. For example, in some of the classrooms sampled, exams were taking place; principals of some of the selected schools declined to participate in the project; or teachers responsible for the class at the time of data collection declined to allow their students to take part. As a result, to obtain the required number of participants, some classrooms and schools were required to substitute subjects for those in the original plan.

## Data analysis

Because age at menarche was normally distributed, we report mean ± standard deviation. The association between age at menarche and year of birth was examined using correlation coefficients and regression models. Age at menarche was stratified into two categories by mean age at menarche from this study which was < 11.8 and ≥ 11.8 years to explore factors associated with having a first period lower than 11.8 years. Potential risk factors were stratified into categories based on the distribution of values or cutoffs of clinical importance. Associations between potential risk factors and age at menarche <11.8 years were assessed first using chi-square tests, then by conducting logistic regression analyses to obtain odds ratios (ORs). Subsequently, factors significantly associated with age at menarche in the univariate analyses were included in the multiple logistic regression models which were type of school, monetary allowance, and year of birth with control for potential confounding factors. In addition, regression analysis of the association between year of birth and age at menarche were performed. Subjects with incomplete data were excluded only for analyses of the variables for which they had missing values. An analysis using indirect age standardization with populations from India [14] and Ghana [15], for which there are reports on age-specific rates of menarche, was also conducted. SPSS Statistics for Windows, Version 22 (IBM Corp., Armonk, NY, USA) was used for data management and regression models. Descriptive analyses, univariate and multivariate analyses were performed using Stata version 13 (Stata Corp., College Station, TX, USA).

## Results

Of the 1,020 participants aged 10–16 years, 537 (52.6%) participants had experienced menarche at the time of data collection. Mean age at menarche was 11.8 ± 1.0 years, with a minimum of 8.2 years and a maximum of 15.0. There was a trend toward a reduction in age at menarche, as shown by mean age at menarche by year of birth (*r* = −0.40, *p* < 0.001), as presented in Table 1. In girls with year of birth from 1997 to 2003, the mean age at menarche decreased; the regression equation obtained was age at menarche = 510.94 − 0.25 × year of birth. Students from schools that are part of Bangkok Metropolis had the lowest mean age at menarche while the highest was found in those from the demonstration school (Table 2). In univariate analyses, the OR for having one’s first period at < 11.8 years in participants born in 2000–2003 was 5.5 times (95% CI: 3.80–8.18) greater than in those born in 1997–1999 (Table 3).

**Table 1:** Age of Thai girls at menarche by year of birth

Year of birth	Age (years)^a^	Number of students		Mean (range)	95%CI
		Total	Number undergoing menarche n (%)		
1997	16	56	54 (96.4)	12.6 (9.8–15.0)	12.3–12.9
1998	15	71	67 (94.4)	12.5 (8.6–14.4)	12.2–12.8
1999	14	93	79 (84.9)	12.1 (10.0–13.9)	11.9–12.3
2000	13	222	165 (74.3)	11.8 (8.2–13.5)	11.7–11.9
2001	12	259	124 (47.9)	11.3 (9.7–12.9)	11.2–11.4
2002	11	235	46 (19.6)	10.8 (9.0–11.7)	10.6–11.0
2003	10	84	2 (2.4)	9.5 (9.0–10.0)	NA^b^
Total		1,020	537 (52.6)	11.8 (8.2–15.0)	11.7–11.9

^a^on Jan 1, 2013; ^b^not available. *CI* confidence interval

**Table 2:** Age of Thai girls at menarche according to type of school

Characteristics	Total n (%)	Number undergoing menarche n (%)	Mean (range)	95%CI
Type of school				
Bangkok Metropolis	116 (11.4)	44 (8.2)	11.4 (9.0–12.7)	11.1–11.6
Ministry of Education school	123 (12.1)	74 (13.8)	11.8 (9.0–14.5)	11.5–12.1
Demonstration school	144 (14.1)	126 (23.5)	12.4 (8.6–15.0)	12.2–12.5
Private school	637 (62.5)	293 (54.6)	11.6 (8.2–14.4)	11.5–11.7

**Table 3:** Social determinants of health-related data for Thai girls experiencing menarche

Characteristics	Age at menarche		Crude OR	95%CI	Adjusted OR	95%CI
	<11.8 n (%)	≥11.8 n (%)				
Total (*n* = 560)	259 (46.3)	301 (53.8)				
Time spent reading (hours/day)						
<1	103 (42.9)	137 (57.1)	1			
1–2.9	143 (49.0)	149 (51.0)	1.3	0.90–1.80		
≥3	13 (46.4)	15 (53.6)	1.1	0.52–2.53		
Watching TV (hours/day)						
<2	131 (44.6)	163 (55.4)	1			
2–3.9	83 (48.0)	90 (52.0)	1.1	0.79–1.67		
≥4	45 (48.4)	48 (51.6)	1.2	0.73–1.86		
Paternal age at birth						
<30.0	41 (42.7)	55 (57.3)	1			
30.0–39.9	157 (47.4)	174 (52.6)	1.2	0.76–1.91		
≥40.0	52 (49.1)	54 (50.9)	1.3	0.73–1.86		
Maternal age at birth						
<30.0	74 (46.0)	87 (54.0)	1			
30.0–39.9	158 (45.5)	189 (54.5)	0.98	0.67–1.43		
≥40.0	17 (58.6)	12 (41.4)	1.7	0.75–3.71		
Body weight (kg)						
<45.0	89 (43.6)	115 (56.4)	1			
45.0–49.9	60 (41.7)	84 (58.3)	0.92	0.60–1.42		
≥50	109 (51.9)	101 (48.1)	1.4	0.95–2.05		
School			*P* < 0.001			
Bangkok Metropolis	24 (9.3)	20 (6.6)	1		1	
Ministry of Education	41 (15.8)	34 (11.3)	1.004	0.47–2.12	1.78	0.77–4.12
Demonstration school	26 (10.0)	116 (38.5)	0.18	0.09-–0.38	0.61	0.26–1.45
Private school	168 (64.9)	131 (41.5)	1.07	0.56–2.01	1.56	0.79–3.08
Playing video games (hours/day)			*P* = 0.015			
<2	160 (61.8)	187 (62.1)	1		1	
2–4	36 (13.9)	64 (21.3)	0.66	0.41–1.04	0.84	0.50–1.40
≥4	63 (24.3)	50 (16.6)	1.47	0.96–2.26	1.75	1.08–2.84
BMI SDS			0.059			
−2 SD to −1 SD	15 (75)	5 (25)	0.21	0.08-0.54		
≤ − 1 SD to +1 SD	251 (57.1)	183 (42.2)	1			
≤ + 1 SD to + 2 SD	35 (44.9)	43 (51.1)	1.64	1.04-2.59		
≥ + 2 SD	15 (53.6)	13 (46.4)	1.04	0.51-2.12		
Monetary allowance (baht/day)			*P* = 0.004			
<50.0	72 (58.1)	52 (41.9)	1		1	
50.0–99.9	151 (44.7)	187 (55.3)	0.58	0.38–0.88	0.8	0.50–1.23
≥100.0	36 (36.7)	62 (63.3)	0.42	0.24–0.72	0.78	0.38–1.28
Year of birth			*P* = 0.001			
1997–1999	50 (22.5)	172 (77.5)				
2000–2003	209 (61.8)	129 (38.2)	5.5	3.8–8.18	5.0	3.6 – 8.0

*BMI SDS* body mass index standard deviation score, *OR* odds ratio

Social determinants of health, such as time spent reading, watching TV, paternal and maternal age at birth, body weight, school type, playing video games, BMI SDS (body mass index standard deviation score), monetary allowance, and year of birth, and their association with age at menarche are shown in Table 3. Body weight, waist circumference, paternal age at birth, maternal age at birth, ฺBMI SDS and time spent reading were not associated with age at menarche statistically significance in the univariate analysis. No associations were found between exercise, food intake and preferences, leisure activities, sleep time, watching television, and having a part time job and age at menarche, using chi-squared tests (data not shown).

The factors significantly associated with age at menarche in the univariate analysis (school type, playing video games, monetary allowance, and year of birth), were analyzed in multivariate regression models to adjust for potential confounders (Table 3). The association between year of birth and age at menarche remained significant in multivariate analysis (*p* = 0.001), as shown in Table 3, while the other factors were not significant.

In an analysis using indirect age standardization for the rate of menarche based on age of the population from Ghana and India, 75.0% of Thai participants aged 11 years had already had their first period by 10 years of age. However, 22.5% of subjects from Ghana aged 11 years old started their first period at 11 years of age, and there was no report of Ghanaian subjects having their first period before or at 10 years. Compared with three age groups of the Indian population, 10.0–11.9, 12.0–13.9 and 14.0–15.9 years, the proportion of students who already experienced menarche at or before 10 years in the Thai population was lower than in populations from India, 8.8% vs. 9.1%, 3.9% vs. 13.1%, and 0.2% vs. 4.6%, respectively.

## Discussion

This study established the current age at menarche in a population-based study in Thai adolescents residing in Bangkok Metropolis. The figure was lower than those previously reported in the United States [3, 4], Europe [2], Canada [18], Ghana [15], and Thailand [8, 9], and found associations between social determinants of health—year of birth and age at menarche. However, different trends were found when comparing the data by indirect age standardization with data from India and Ghana. A trend toward a younger age at menarche by stratifying by year of birth in this study was similar to findings reported by other studies [2–4, 8, 9, 19]. Lifestyle and environmental factors, including changes in time spent reading, using social networking services, sleeping, and watching television, and changes in nutritional status, are possible explanations for the decrease in age at menarche that have been proposed in a number of studies [12, 19] and for the substantial change over a short time period in this study’s finding. The lifestyle and environmental changes’ impact on age at menarche may have been influenced by disturbing the hormonal axis, which will be discussed later.

In Thailand, particularly in urban areas such as Bangkok, increased rates of obesity reflect high caloric consumption among adolescents in the region, as reported in a recent review article [19]. In the current study, there was no association between BMI SDS and age at menarche. These findings differed from those reported in studies conducted in Hong Kong [20] and Canada [18], which found an inverse association between BMI and age at menarche. For example, Qing et al. reported that in Hong Kong, each unit increase in BMI was associated with a decrease of 0.7 years in the age of menarche [20]. However, Qing et al. assessed the association between BMI and age at puberty onset using a continuous BMI variable; this approach differed from the one taken in the present study, which analyzed BMI as a BMI SDS and a categorical variable. The majority of participants in the present study had normal BMI SDS, and the group with BMI SDS ≥ +2 SD included only 28 students. This could have affected the precision of our analyses. In addition, it is known that BMI varies by race and ethnicity, which could be another explanation for the differences [21].

There have also been reports on age at menarche and other anthropometrical measurements, such as weight, height, and weight for height. In 1977, Rose E. Frisch investigated the relationship between anthropometric factors and age at menarche, hypothesizing that an increase in weight for height, indicating improved nutritional status, would decrease age at menarche [22]. In the present study, it was hypothesized that nutritional status was also related to social determinants of health. Thus, the monetary allowance was used to indirectly assess both nutritional status and social determinants of health. It is believed that nutritional status influences age at menarche via the neuroendocrine system, and in particular the hypothalamus-pituitary-gonadal (HPG) axis, thus linking lifestyle changes to age at menarche. The HPG axis affects pubertal onset via its pulsatile release of luteinizing hormone, gonadotropin-releasing hormone and kisspeptin [23]. This might not be true for our population in terms of nutritional status, in which a higher monetary allowance was associated with reduced risk of experiencing menarche at < 11.8 years as found in the univariate analysis even though there was no association in the multivariate analysis. This suggests that when investigating early age at menarche, race and ethnicity should be considered. However, lifestyle changes in the urban area may be an influential factor which has not been clearly proved from this study.

There was no association between parental age at birth and age at menarche in this study, in contrast to a 2010 report from the United States [24]. In that study, age at menarche was found to decrease with every 1 year increase in the age of the parents [24]. This may be related to race and ethnicity differences. The effect of parental age on age at menarche has been explained by endocrinological irregularities in daughters of mothers of advanced maternal age [25] and a reduction in reproductive fitness among female offspring because of delayed parenthood [26].

This study had some limitations. One weakness was the generalizability. The study intended to include students from all socioeconomic classes in Bangkok as a result of our school-based sampling strategy to be representative of preteens and teens across the Bangkok urban area. However, the major limitation in the present study was the unforeseen problems that arose during fieldwork, preventing the authors from adhering to the original protocol. Unfortunately, other variables that could potentially influence age at menarche, such as genetic factors, could not be explored. In addition, to keep questionnaires to a reasonable length, some lifestyle factors of interest could not be examined. Additionally, there may be some information biases from recalled data by participants. Both may be causes of systematic errors. However, the target number of participants was reached, allowing the authors to maintain the statistical power desired.

## Conclusions

The mean age at menarche of female adolescents in Dusit District, Bangkok, Thailand was 11.8 ± 1.0 years. This was lower than reported in previous studies, and age at menarche was correlated with year of birth (*r* = −0.40, *p* < 0.001). Type of school, monetary allowance, and year of birth were related to age at menarche in the univariate analyses. However, only year of birth was associated with age at menarche in the multivariate analysis.

## Abbreviations

BMA: Bangkok Metropolitan Administration
BMI: Body mass index
CDC: Center for Disease Control and Prevention
CIs: Confidence interval
